# Food Insecurity during COVID-19 in Yemen

**DOI:** 10.4269/ajtmh.22-0059

**Published:** 2022-06-15

**Authors:** Zainab Syyeda Rahmat, Zarmina Islam, Parvathy Mohanan, Diana Mutasem Kokash, Mohammad Yasir Essar, Mohammad Mehedi Hasan, Hashim Talib Hashim, Ashraf Fhed Mohammed Basalilah

**Affiliations:** ^1^ Faculty of Medicine, Dow Medical College, Dow University of Health Sciences, Karachi, Pakistan;; ^2^ Faculty of Medicine, Medical University Sofia, Sofia, Bulgaria;; ^3^ Dubai Health Authority, Dubai, United Arab Emirates;; ^4^ Kabul University of Medical Sciences, Kabul, Afghanistan;; ^5^ Department of Biochemistry and Molecular Biology, Faculty of Life Science, Mawlana Bhashani Science and Technology University, Tangail, Bangladesh;; ^6^ University of Baghdad, College of Medicine, Baghdad, Iraq;; ^7^ Hadhramaut Hospital, Hadhramaut, Yemen

## Abstract

The United Nations has declared Yemen as the world’s worst humanitarian crisis with 21 million people in need of humanitarian assistance. Due to the convergence of severe economic instability exacerbated by the COVID-19 pandemic, stifling war, and spiking food prices, the Yemeni people are at the brink of famine with women and children especially malnourished. Desperate to feed their families, civilians are forced to resort to begging, participate in child marriages, or plunge into debt. An inflated currency has significantly diminished the purchasing power of the Yemeni population, and COVID-19 restrictions have made acquisition of food and essential commodity imports arduous. Immediate action by global and local governments is essential to prevent the deaths of thousands of people in the wake of severe food scarcity.

## INTRODUCTION

Yemen has long been ranked as the poorest country of the Arabian Peninsula, with almost 80% of the population residing under the poverty line.^1^ With a gross domestic product of 23.49 billion USD in 2018, Yemen contributes 0.02% of the world economy.^2^ The United Nations (UN) has detailed that since 2015, more than 3.6 million Yemeni citizens have been displaced due to ongoing conflict, insecurity, natural disasters, and COVID-19, which has recently emerged as a significant cause of displacement.^3^^,^^4^ Despite an ongoing civil war, the UN has projected that since 2015, 131,000 of the estimated 233,000 deaths in Yemen have been related to causes other than armed conflict, such as food insecurity.^5^

Food insecurity is specified as the lack of money or resources interfering with food intake, and key contributors to food insecurity include lack of employment, poverty, and low income.^5^^–^^7^ In Yemen, 16.2 million people, which accounts for approximately 45% of the population, are food insecure, even after humanitarian assistance.^8^ Taking a closer look, 11 million out of 16.2 million people have approached “crisis” levels of food insecurity, and approximately 47,000 people have been pushed into absolute famine.^8^

Before the COVID-19 pandemic, multiple converging factors including political conflict, devaluing currency, lack of global aid, and natural disasters mitigated the food insecurity crisis in Yemen. The emergence of COVID-19 and its related restrictions on trade have worsened the conditions and play a major role in exacerbating it. These factors caused more than 50% of Yemen’s population to face hunger in 2021.^9^ For example, a study conducted by the Norwegian Refugee Council found that since the beginning of the coronavirus pandemic, more than three-quarters of displaced Yemeni families have lost all their income.^10^ In this article, our aim is to discuss the double burden Yemen bears in fighting the COVID-19 pandemic and dealing with a worsening acute food crisis, both of which are eviscerating the already war-torn country.

## CHALLENGES

Approximately 113 million people worldwide were severely food insecure before the pandemic hit, as stated by the Food and Agriculture Organization.^11^

One major instigator of Yemen’s food crisis is political instability, which caused the permanent closure of Yemen’s air, ocean, and land ports in 2018, locking in 27 million individuals, and keeping out 500,000 metric tons of essential resources such as fuel and food.^12^ The ports were revived only for limited humanitarian help. The repercussions of this closure were felt in less than 24 hours as fuel prices increased as much as 60% in some areas.^12^ Because Yemen imports around 90% of its food, the closure of ports has resulted in a conspicuous soaring of food costs and put a predictable strain on the availability of food, significantly increasing the chance of large-scale starvation in Yemen.^13^ If political conflict in Yemen continues through 2022, the UN warns that the country, with a population of 29.83 million, will become the poorest in the world.^1^

In addition, devaluation of the Yemeni riyal have led to inflated food prices since 2015.^14^ Due to the exhaustion of foreign trade reserves, Yemeni local currency is tremendously inflated, and 19% of its value has been lost against the U.S. dollar within the first half of 2020, outperforming the levels noted in the 2018 crisis.^15^ This has led to soaring prices, yet a diminished purchasing power of Yemeni citizens.^16^ For more than 1 year, more than 1.2 million Yemeni government employees have not received their salaries.^16^ High levels of inflation directly correlates to reduced purchasing power, which is pushing more Yemenis into poverty according to a 2020 report.^17^ In areas of Yemen operated by the internationally recognized government where Yemeni currency continues to devalue, the normal cost of the smallest food basket increased by 7% in August 2021.^18^ Indeed, before the pandemic, almost 80% of Yemenis were dependent on humanitarian help.^9^ However, the UN has announced a reduction in food aid to Yemen due to lack of funds.^19^ Starting in January 2022, the World Food Program (WFP) has detailed that 8 million Yemenis will be given “barely half” of the agency’s daily minimum allocation.^19^ The agency detailed that it needs more than a billion dollars in 2022 to deliver food assistance to starving families in Yemen.^20^ Another important factor contributing to rising food prices is constant flooding. In October 2008, April 2020, and again in July 2021, floods continuously damaged private and public property, as well as food stocks.^21^^,^^22^

To make matters worse, Yemen is particularly vulnerable to the COVID-19 pandemic due to a crumbling healthcare system.^11^ As of March 2022, there have been a total of 11,783 confirmed cases of coronavirus with 2,139 deaths in Yemen, according to the WHO.^23^ The actual number of cases is expected to be much higher because a dysfunctional healthcare system and war-related instability has led to inadequate testing capacity.^9^ Catastrophic socioeconomic consequences have resulted from various COVID-19 pandemic-related restrictions in a country already engulfed in civil war, including spiraling food insecurity.^9^ Even though Yemen is a country in which 80% to 90% of food must be imported, comparison between food imports in March of 2019 and March 2020 show a subsequent decrease by 43% due to coronavirus-related restrictions, as observed by the WFP.^9^^,^^24^ Ninety-four percent of families surveyed by the Norwegian Refugee Council reported that prices of food and other items have risen since the COVID-19 outbreak.^25^ In a country where only 1.2% of the population is fully vaccinated, outbreak of SARS-CoV-2 is a looming threat that will disproportionately affect malnourished people who lack access to clean water and sanitation.^11^^,^^26^

The consolidation of various challenges confronted by Yemen, including constant turmoil due to the civil war, the continuous decline of humanitarian aid, less effective supply chain systems (due in part by COVID-19 related restrictions), and rising food prices have propagated Yemen’s vulnerability to starvation.^9^ Inflated food costs and decreased purchasing power remain noteworthy concerns for millions of vulnerable family units.

## IMPLICATIONS OF FOOD INSECURITY IN YEMEN

An important implication of widespread food insecurity in Yemen is the related poor health outcomes. Cholera, for example, has been rampant in Yemen since its outbreak in 2016.^27^ The WHO reported more than 320,000 cases of cholera in 2017 and 1,700 victims, with the suspected cases to reach one million in 2018.^27^ At one point, it had become the largest outbreak worldwide in recorded history.^28^ When inspecting these cases more closely, a significant correlation between food insecurity and ill health is established by the fact that most children who were infected with cholera were initially weakened by malnourishment.^27^ Diminishing global aid is depriving many Yemenis of the food they need, which is predisposing them to infections such as cholera.^29^

Other health-related implications of food insecurity have been observed in many countries, including multiple sub-Saharan African countries, such as Mali, Niger, and Sudan, as well as Yemen.^30^^,^^31^ These countries have an increased case-fatality ratio for COVID-19, rank high on at least three malnutrition indicators, and show a moderate positive association with iron deficiency, highlighting how long-term food insecurity is related to ill health.^30^ Additionally, in one Yemeni study, measles incidence was reported to be three times higher among severe acute malnutrition children, and diarrhea, fever, and cough were also significantly higher.^32^ Emerging conflicts, severe contraction of the economy, and COVID-19 will not only burden the healthcare system but also exacerbate preexisting socioeconomic disparities and further contribute to mortality, especially in children.^32^ Long-term impacts include impaired learning and cognitive development in children, maternal depressive disorders, chronic conditions such as asthma, and suicidal ideation.^33^

On the extreme, some Yemeni families have resorted to child marriages, child labor, or begging to fulfill their dietary needs.^34^

Taken together, the short- and long-term implications of food insecurity are vast, hence immediate humanitarian relief efforts are necessary to reduce the likelihood of such complications. People around the world, including healthcare professionals and scientists, will also benefit by the alleviation of food insecurity because outbreaks of infection may lead to increased drug resistance, as seen with the rise in extensively resistant strains of typhoid bacteria.^35^ A similar development can arise in Yemen if this food crisis is not resolved, including the development of drug-resistant strains of cholera and other organisms.

## EFFORTS AND RECOMMENDATIONS

Several efforts have been made to mitigate the food crisis, with a focus on boosting the economy, provision of basic needs to disadvantaged communities in rural areas, and efforts to balance health measures and economic ramifications. Efforts have been implemented in Yemen in the past; however, several barriers continue to exist, especially in the wake of the SARS-CoV-2 outbreak and the presence of political instability.

The WFP of the UN has been delivering food relief on the ground in Yemen, providing food and financial assistance for 13 million people.^36^ In response to serious malnutrition rates among children, the WFP is offering dietary support to 3.3 million pregnant women and children under age 5.^36^ However, the WFP halved funding to some parts of Yemen only a day before the first coronavirus case was announced in March 2020.^37^

Food security and agriculture cluster partners ramped up humanitarian assistance significantly in 2019, driving noteworthy food security improvements, with the number of people assisted increasing by 47% from an approximate of 2.48 million in December 2018 to 3.64 million within a year.^38^ However, since the escalation of political turmoil and misuse of funds, the United States as well as international organizations announced the suspension of its humanitarian assistance in March 2020, which was crucial to combat the spread of COVID-19.^37^^,^^39^ Yet significant improvements were achieved as aid agencies managed to provide food to 13 million Yemenis every month in 2021.^40^

Despite several efforts made to combat Yemen’s hunger crisis, 16.2 million Yemenis are food insecure.^36^ The first step in achieving relief for Yemenis from the acute food insecurity crisis is resolving the debilitating conflict. Therefore, pressure must be placed on international powers to help end the conflict in Yemen before the citizens approach famine. Lifting bans and restrictions placed on Yemen’s ports to accelerate the movement of goods and therefore ease prices and providing unrestricted access to essential resources are also vital steps in solving this crisis. If this were to take place, not only would Yemen’s food insecurity crisis resolve, but its associated factors such as poor health outcomes would also be avoided. Urgent action is needed from local powers to address the coronavirus pandemic, which itself is hindering any attempts made toward attaining humanitarian assistance. It is vital to provide the country with urgent funding to ensure continued and unrestricted assistance to save lives while paying particular attention to displaced people and vulnerable populations that are hit the hardest by the food insecurity.
